# Effects of Different Packaging Materials on Egg Translucency, Quality, and Shell Surface Microbiota

**DOI:** 10.3390/foods15071255

**Published:** 2026-04-07

**Authors:** Yihan Wang, Quanzhong Wei, Zeyao Zhang, Lin Xuan, Jiajie Yang, Mimi Lei, Tingting Liang, Xuefeng Shi

**Affiliations:** 1 College of Animal Science and Technology, Guangxi University, Nanning 530004, China; wyh030714@163.com (Y.W.); 19353547516@163.com (Z.Z.); 15878370757@163.com (M.L.); 18978024406@163.com (T.L.); 2 College of Animal Science and Technology, China Agricultural University, Beijing 100193, China; 13517712801@163.com (Q.W.); linxuan@cau.edu.cn (L.X.); yjiae@cau.edu.cn (J.Y.)

**Keywords:** packaging materials, egg translucency, egg quality, eggshell microbiota, food safety

## Abstract

Egg quality during storage is a critical factor influencing consumer acceptance and food safety. However, the effects of storage methods on eggshell translucency and surface microbiota remain insufficiently understood. In this study, three common packaging methods, paper pulp trays (PPT), expanded polyethylene foam trays (EPE), and transparent plastic boxes (TPB), were evaluated to assess their impact on egg translucency, internal quality, and microbial communities. Egg quality traits were measured, and microstructural and elemental characteristics were examined using scanning electron microscopy and compositional analysis. In addition, 16S rRNA sequencing was performed to characterize the eggshell surface microbiota. The packaging method significantly influenced translucency development, with EPE mitigating mottling better than PPT and TPB. Storage duration was the predominant driver of internal quality deterioration, particularly affecting the albumen height and Haugh units. Translucency was not associated with shell thickness or mineral content but was likely associated with moisture dynamics. Distinct microbial communities are shaped by different packaging materials. These findings provide new insights into the mechanisms underlying translucency and microbial ecology during egg storage. This highlights the practical implications of optimizing packaging strategies to maintain egg quality, extend the shelf life, and ensure microbial safety.

## 1. Introduction

Eggs are an inexpensive and high-quality source of nutrients, particularly protein, iron, vitamins, and phosphorus. They play an important role in improving dietary structure [1,2,3]. They are also one of the most widely consumed animal-derived foods worldwide [4,5]. Nevertheless, egg quality deteriorates during storage, involving external appearance and internal attributes. This subsequently diminishes consumer acceptance and market value [6,7,8]. Therefore, storage practices and packaging materials are critical egg quality determinants [8,9].

With the continuous improvement in living standards, consumers have become increasingly demanding of high-quality eggs [10]. Purchasing behavior is predominantly shaped by factors such as price, sensory characteristics, and nutritional value [11]. Because fresh eggs account for most consumption, their external appearance is a critical determinant of consumer preference. Also referred to as mottling, egg translucency is a common defect in egg production and is characterized by grayish spots visible under illumination [12]. It is mainly attributed to abnormal moisture content and microstructural alterations in the eggshell [12,13,14]. Shi et al. [4] reported that translucent eggs were significantly associated with the mastoid space height, mastoid space width, mastoid space area, and eggshell membrane thickness. Xuan et al. [15] found that the translucent region has reduced shell strength, elevated moisture content, and increased matrix protein levels, impairing moisture regulation and gas exchange. Three-dimensional micro-CT analysis showed abnormally narrow pores and irregular structures in the translucent regions, resulting in moisture retention and impaired gas exchange. This further promoted translucency formation. Factors such as the breed and age of the hen, diet composition, and environmental conditions, such as temperature and humidity, can influence translucency [4,14,16,17].

Translucent eggs also have varying degrees of impact on internal quality [18,19]. Chousalkar et al. [13] suggested that structural changes in the mammillary and palisade layers during translucency development increased the likelihood of shell cracks. This facilitates the penetration of pathogens such as *Salmonella* and *E. coli*. However, Shi et al. [4] reported contrasting results, showing that eggshell translucency did not increase the risk of *E. coli* trans-shell invasion. Therefore, the effect of translucency on egg safety remains controversial. No significant association was observed between egg translucency and internal egg quality [20].

The eggshell surface has complex microbial communities that play important roles in determining egg safety and internal quality [6,9,21]. Although the impacts of temperature and washing have been widely investigated, the effects of storage methods on eggshell microbiota remain unclear. Elucidating these microbial dynamics is critical for developing effective preservation strategies and for maintaining egg freshness and safety.

In this study, the effects of three common storage methods, namely PPT, EPE, and TPB, on egg translucency, quality traits, and eggshell surface microbiota were evaluated. Integrating egg quality assessment, scanning electron microscopy, elemental analysis, and 16S rRNA sequencing increases our understanding of egg translucency and microbial dynamics during storage, offering practical guidance for enhancing egg quality and microbial safety. A storage temperature of 25 °C was selected to better simulate real-world conditions commonly encountered during egg distribution.

## 2. Materials and Methods

### 2.1. Animals and Samples

A total of 300 healthy Jingfen No. 6 laying hens (40 weeks of age, body weight 1.50 ± 0.50 kg) were obtained from a commercial laying hen farm in Nanning, Guangxi, China. The hens were housed individually in battery cages (one hen per cage) within the same facility. All the hens were maintained under identical management conditions with automated temperature control, 16 h of artificial light, 8 h of darkness, and a uniform diet throughout the laying period. All the hens were confirmed to be in good health.

Fresh eggs were collected simultaneously, and abnormal eggs (dirty, cracked, broken, deformed, undersized, or oversized) were excluded. In total, 210 eggs were used in this study. Translucency grading was conducted immediately after collection, and 30 eggs were randomly selected for initial quality measurements. The remaining 180 eggs were randomly assigned to one of the three storage treatments: paper pulp trays (PPT), expanded polyethylene foam trays (EPE), or transparent plastic boxes (TPB).

### 2.2. Experimental Design

**Figure 1:**
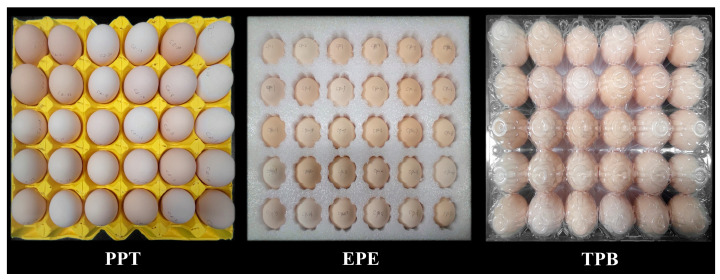
Three egg storage methods. PPT, paper pulp tray; EPE, expanded polyethylene foam tray; TPB, transparent plastic box.

The experimental grouping design is illustrated in Figure 1. Eggs were stored in PPT, EPE, or TPB at constant temperature (25 °C) and relative humidity (50%) conditions for 21 d in a constant temperature and humidity incubator (Model HWS-250, Ningbo Jiangnan Instrument Factory, Ningbo, China).

### 2.3. Grading of Translucent Eggs

**Figure 2:**
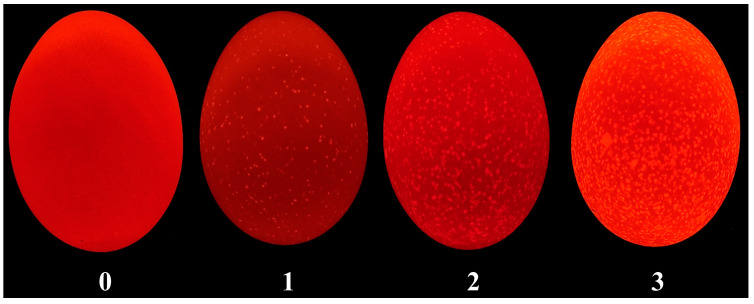
Translucent egg grading pattern diagram. From left to right, eggs are assigned scores of 0, 1, 2, and 3. Grade 0 represents excellent quality, with only a few slightly bright spots under LED light and no black spots under natural light. Grade 1 represents mild translucency, with several bright spots under LED light. Grade 2 indicates moderate translucency, with many bright spots distributed across the shell under LED light. Grade 3 indicates severe translucency, with dense small and large bright spots over the entire shell surface under LED light.

During the first three days of storage (0, 24, 48, and 72 h), the translucency of eggs was evaluated. Based on the size and density of the translucent spots, four representative eggs were selected as reference samples and assigned scores from 0 to 3 (Figure 2) [4]. At each time point, the translucency of 60 eggs from each group was examined. The grading was performed in a dark room. Under dark conditions, the eggs were illuminated with an LED light (Model YX-618, Shenzhen Yuxin Electronics Co., Ltd., Shenzhen, China; 6000 K, ~1500 lux at the egg surface, measured with a digital lux meter (BULL, Beijing, China)) and scored according to the reference samples. To minimize random errors, three trained evaluators scored the eggs independently. A score was considered valid only when at least two evaluators agreed. Otherwise, the egg was re-evaluated and regraded.

### 2.4. Determination of Egg Quality

In addition to the 30 eggs assessed at the baseline, egg quality measurements were performed on days 7, 14, and 21 of storage, with ten eggs randomly selected from each group at each time point. The measured parameters included egg weight (EW), egg shape index (ESI), eggshell strength (ESS), Haugh unit (HU), albumen height (AH), and yolk color (YC). A multifunctional egg quality tester (EMT-5200; Robotmation, Tokyo, Japan) was used to measure the EW, HU, AH, and YC. Eggshell strength was determined using a Model-II eggshell strength tester (Robotmation, Tokyo, Japan). Eggshell thickness was measured at three locations at the bottom, middle, and top using a micrometer screw gauge, and the mean value was calculated.

### 2.5. Determination of Eggshell Ultrastructure and Elemental Composition

After 3 d of storage, 10 eggs were selected to examine translucent eggs (TE) and non-translucent eggs (NTE). Eggshell samples were further divided into translucent (TA) and non-translucent (NTA) areas for analysis. Eggshell ultrastructure, including total thickness (TT), effective layer thickness (ET), mammillary layer thickness (MT), and eggshell membrane thickness (EMT), and elemental composition (Ca, P, Na, S, Mg, and C), was analyzed using scanning electron microscopy (SEM, SU8020, Hitachi, Tokyo, Japan).

Samples were prepared as described by Zhao [22] with minor modifications. Eggshell fragments of approximately 1 cm^2^ were dried, mounted on a conductive adhesive tape, and sputter-coated with gold using a Quorum SC7620 sputter-coater (Quorum Technologies Ltd., Laughton, UK; 45 s, 10 mA). Morphological observations were conducted using a TESCAN MIRA LMS scanning electron microscope (TESCAN, Brno, Czech Republic) at 3 kV, and energy-dispersive spectroscopy (EDS) mapping was performed at 15 kV using an SE2 secondary electron detector (TESCAN, Brno, Czech Republic).

### 2.6. Bacterial Sampling of Surface of Eggshells

On day 21 of storage, 18 eggs were randomly selected from each group. Sterile cotton swabs moistened with physiological saline were used to wipe the entire egg surface. Swabs from three eggs were pooled into a 5 mL centrifuge tube, yielding six composite samples per group. All the samples were immediately stored at −80 °C for microbial community analysis.

### 2.7. Microbial DNA Extraction and 16S rRNA Amplicon Sequencin

Microbial genomic DNA was extracted from eggshell surface samples using a Stool DNA Kit (Omega Bio-tek, Norcross, GA, USA), following the manufacturer’s instructions. DNA integrity was verified by 1% agarose gel electrophoresis, and the concentration and purity were determined using a NanoDrop 2000 UV–Vis spectrophotometer (Thermo Scientific, Wilmington, DE, USA).

The hypervariable V3–V4 region of the bacterial 16S rRNA gene was amplified with primers 343F (5′-TACGGRAGGCAGCAG-3′) and 798R (5′-AGGGTATCTAATCCT-3′) using an ABI GeneAmp 9700 PCR thermocycler (Applied Biosystems, Foster City, CA, USA). The PCR protocol consisted of an initial denaturation at 95 °C for 3 min; 27 cycles of denaturation at 95 °C for 30 s, annealing at 55 °C for 30 s, and extension at 72 °C for 45 s; and a final extension at 72 °C for 10 min, followed by holding at 4 °C.

Each 20 μL PCR mixture contained 4 μL of 5× TransStart FastPfu buffer, 2 μL of 2.5 mM dNTPs, 0.8 μL of forward primer (5 μM), 0.8 μL of reverse primer (5 μM), 0.4 μL of TransStart FastPfu DNA Polymerase, 10 ng of template DNA, and nuclease-free water to volume. PCR was performed in triplicate. The products were separated on 2% agarose gels, purified using the AxyPrep DNA Gel Extraction Kit (Axygen Biosciences, Union City, CA, USA), and quantified using a Quantus Fluorometer (Promega, Beijing, China). The methods used for DNA extraction and 16S rRNA amplicon sequencing were adapted from standard protocols widely used in microbiome studies [23].

### 2.8. Bioinformatic Analysis

Library preparation, sequencing, and initial data processing were performed by OE Biotech Co., Ltd. (Shanghai, China). Raw FASTQ reads were processed with Cutadapt to remove adapters and then filtered, denoised, merged, and screened for chimeras using the DADA2 plugin in QIIME2 (version 2020.11) [24,25]. Representative sequences and an amplicon sequence variant (ASV) abundance table were generated. Taxonomic classification was conducted using the SILVA database (version 138) and the QIIME2 q2-feature-classifier plugin.

Alpha diversity indices (ACE, Chao, Shannon, and Simpson) were calculated using QIIME2 [26]. Beta diversity was evaluated using unweighted UniFrac distances, and principal coordinate analysis (PCoA) was conducted in R to visualize community differences. Genus-level co-occurrence networks were constructed based on Spearman’s correlation, with correlations considered significant at |R| > 0.8 and *p* < 0.05. Rank–abundance curves were generated using the rankabund function in R (version 2.6.2) to assess sequencing depth. The relative abundance was analyzed across multiple taxonomic levels.

Genus-level Venn diagrams were used to compare the microbial compositions among the PPT, EPE, and TPB groups. Differential taxa from phylum to genus were identified using linear discriminant analysis effect size (LEfSe) with a threshold of LDA score > 3.0 and *p* < 0.01 [27]. Ternary plots were generated using the R software (version 4.4.3). Functional prediction of microbial pathways was conducted using PICRUSt2 based on 16S rRNA gene sequences and annotated against the Kyoto Encyclopedia of Genes and Genomes (KEGG) database.

### 2.9. Statistical Analysis

All the statistical analyses and data visualizations were performed using the R software (version 4.4.3). The mean translucency score and egg quality parameters were analyzed using one-way analysis of variance (ANOVA). The results are presented as mean ± standard error of the mean. Differences were considered statistically significant at *p* < 0.05.

## 3. Results

### 3.1. Effect of Different Storage Methods on Egg Translucency

**Figure 3:**
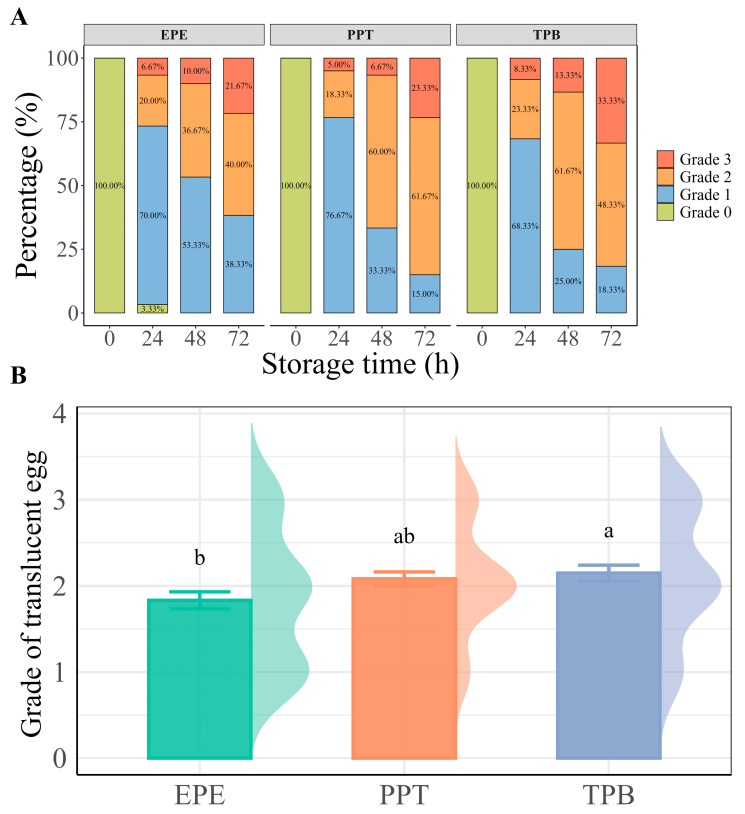
Effect of storage method and time on egg translucency. (**A**) Changes in translucency grades of eggs under three storage methods over time; (**B**) Mean translucency scores of eggs after 72 h under different storage conditions. Different letters above the bars indicate significant differences among groups (*p* < 0.05).

Changes in the translucency grades of eggs under different storage conditions are shown in Figure 3A. At the beginning of storage (0 h), all the eggs in the three groups were classified as grade 0, and no translucency was observed. As the storage time progressed, translucency gradually increased in all groups, with a higher proportion of eggs reaching more severe grades. At 72 h, clear differences were observed among the storage methods. The proportions of grade 2 and 3 eggs were higher in the PPT and TPB groups than in the EPE group. This indicates that EPE storage has a suppressive effect on translucency development.

Mean translucency scores at 72 h further confirmed this finding (Figure 3B). Eggs stored in EPE trays had a significantly lower translucency score (1.83 ± 0.10) than those stored in TPB (2.15 ± 0.10, *p* < 0.05). The mean translucency score of the PPT group (2.08 ± 0.08) did not differ significantly from either the EPE or TPB groups. Therefore, the EPE packaging protects against translucent development.

### 3.2. Effect of Translucency on Eggshell Physical Characteristics

**Figure 4:**
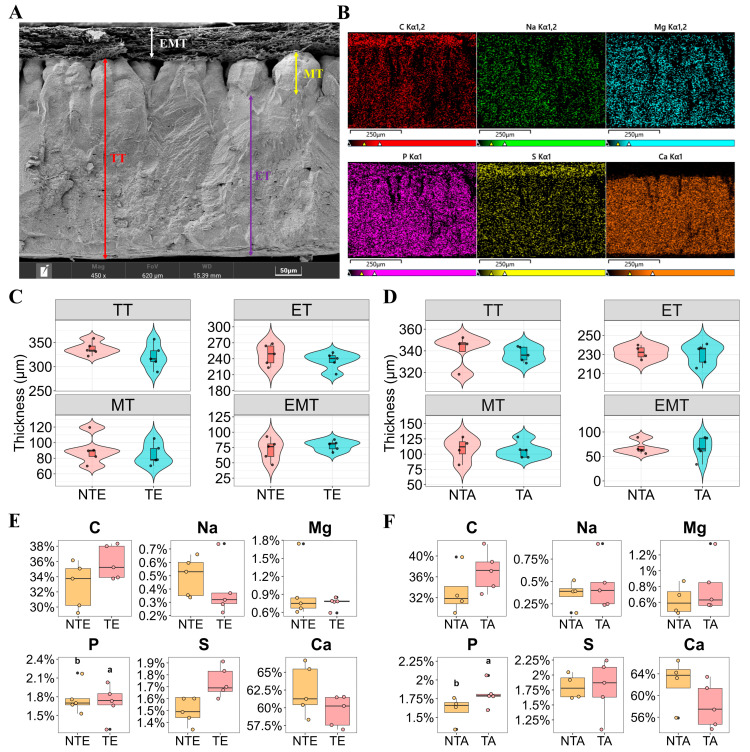
Effect of translucency on eggshell physical characteristics. (**A**) Scanning electron microscopy (SEM) micrographs showing the cross-sectional morphology of the eggshell; (**B**) Elemental distribution maps illustrating the spatial distribution of C, Na, Mg, P, S, and Ca using color-coded mapping; (**C**) Comparison of elemental percentage composition between TA and NTA within the same egg; (**D**) Comparison of eggshell microstructural parameters between TE and NTE; (**E**) Comparison of eggshell microstructural parameters between TA and NTA within the same egg; (**F**) Comparison of elemental percentage composition between TE and NTE. TT, total thickness; ET, effective layer thickness; MT, mammillary layer thickness; EMT, eggshell membrane thickness; NTE, non-translucent egg; TE, translucent egg; NTA, non-translucent area; TA, translucent area.

To further investigate the factors contributing to differences in eggshell translucency under different storage conditions, we examined eggshell microstructure and elemental composition after 3 d of storage by comparing TE with NTE, and translucent areas (TA) and non-translucent areas (NTA) within the same egg (Figure 4). Scanning electron microscopy (SEM) micrographs (Figure 4A) showed the cross-sectional morphology of the eggshell. Meanwhile, elemental mapping (Figure 4B) illustrated the spatial distribution of the major elements (C, Na, Mg, P, S, and Ca) using color-coded maps. Within the same egg, no significant differences (*p* > 0.05) were detected between TA and NTA in terms of elemental percentage composition (Figure 4C). No significant differences (*p* > 0.05) were observed between TE and NTE in eggshell microstructural parameters (TT, ET, MT, or EMT) (Figure 4D). Within the same egg, no significant differences (*p* > 0.05) were detected between TA and NTA regarding microstructural parameters (Figure 4E). The elemental percentage composition (Na, Mg, P, S, C, and Ca) did not differ significantly between TE and NTE (*p* > 0.05) (Figure 4F).

### 3.3. Effects of Different Storage Methods on Egg Quality

The effects of different storage methods on egg quality parameters are presented in Table 1. On day 0, baseline values of egg quality traits were recorded. No significant differences (*p* > 0.05) were observed among the three storage methods (EPE, PPT, and TPB) in terms of ESI, ESS, EW, YC, AH, and HU.

As shown in Table 1, with prolonged storage, egg quality gradually declined, regardless of the storage method. EW showed an initial increase on day 14, particularly for the EPE and PPT groups, followed by a decrease on day 21. This increase in EW on day 14 could be attributed to slight variations in moisture content or temporary changes in storage conditions, which might have affected the weight of the eggs. Meanwhile, albumin height and HU decreased after 14 d of storage. YC exhibited slight fluctuations without significant differences among the groups. Eggshell strength and thickness remained relatively stable throughout storage. The data in Table 1 also indicate that different storage methods did not have significant effects on egg quality parameters under the same environmental conditions. Meanwhile, storage duration was the main factor contributing to quality deterioration, especially in AH and HU.

### 3.4. Effects of Storage Methods on Eggshell Surface Microbial Diversity and Community Structure

**Figure 5:**
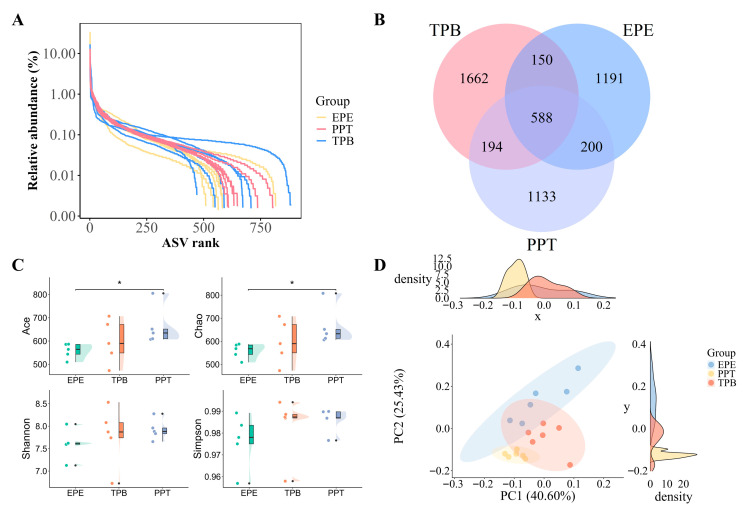
Diversity of eggshell surface microbiota under different storage methods. (**A**) Rank–abundance curves showing ASV richness and evenness across groups; (**B**) Venn diagram illustrating shared and unique ASVs among EPE, TPB, and PPT groups; (**C**) Alpha diversity indices (Ace, Chao, Shannon, and Simpson) of microbial communities (* *p* < 0.05); (**D**) Principal coordinate analysis (PCoA) based on Bray–Curtis distance showing separation of microbial community structures among groups.

High-throughput sequencing of 18 samples produced 1,432,989 paired-end reads, yielding 1,322,756 clean reads after quality control (≥65,951 per sample; average 73,486). Rank–abundance curves (Figure 5A) showed long tails, indicating many low-abundance ASVs with good community evenness. Venn analysis (Figure 5B) identified 5118 ASVs, including 588 shared among all groups and 1191, 1662, and 1133 unique to EPE, TPB, and PPT, respectively, revealing distinct microbial compositions. Alpha diversity (Figure 5C) showed higher ACE and Chao indices in PPT than EPE (*p* < 0.05), while TPB was intermediate; Shannon and Simpson indices showed no significant differences (*p* > 0.05) among the groups. PCoA (Figure 5D) revealed clear separation among groups, with PC1 and PC2 explaining 40.6% and 25.4% of the variance, confirming distinct microbial community structures.

### 3.5. Taxonomic Composition and Differential Microbial Groups Across Storage Methods

**Figure 6:**
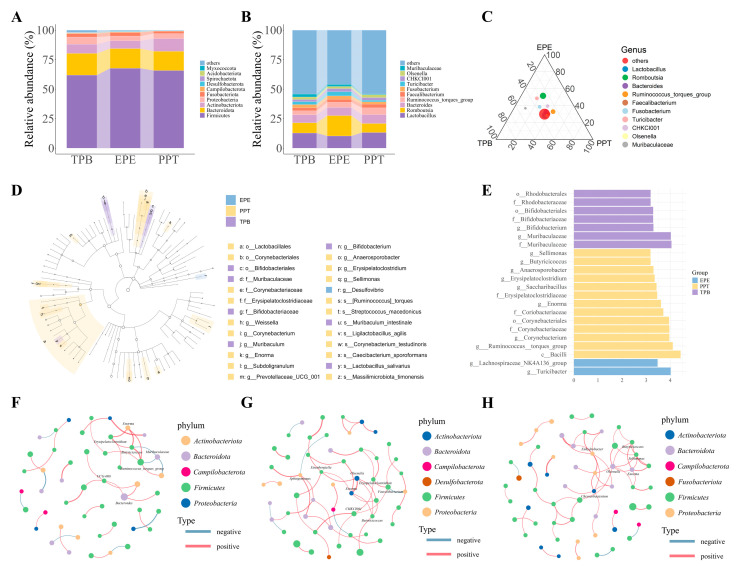
Microbial community composition and differential taxa of eggshell surface microbiota. (**A**,**B**) show the relative abundances of bacterial phyla and genera, respectively. Relative abundances of the top ten taxa are shown; remaining taxa are combined as “Others”; (**C**) Ternary plot illustrating the distribution of dominant genera among the three storage methods; (**D**) Lefse cladogram showing taxa with significant differences at multiple taxonomic levels (LDA > 3.0). (**E**) Histogram of LDA scores for significantly enriched taxa (LDA > 3.0); (**F**–**H**) Co-occurrence networks of the top 100 genera in EPE, TPB, and PPT groups, respectively. Node size reflects relative abundance; colors represent different phyla; red and blue edges indicate positive and negative correlations (Spearman |R| > 0.8, *p* < 0.05). k, kingdom; p, phylum; c, class; o, order; f, family; g, genus; s, species.

Figure 6A,B depict the relative abundance of the eggshell surface microbiota at the phylum and genus levels, respectively. At the phylum level (Figure 6A), the eggshell microbiota across all groups was dominated by *Firmicutes*, *Bacteroidota*, *Actinobacteriota*, *Proteobacteria*, and *Fusobacteriota*, together accounting for more than 96% of the total sequences. The eggshell surface microbiota in the TPB, EPE, and PPT groups were dominated by five bacterial phyla: *Firmicutes* (61.88%, 67.69%, and 65.80%, respectively), *Bacteroidota* (18.36%, 16.67%, and 16.23%, respectively), *Actinobacteriota* (7.78%, 6.67%, and 10.71%, respectively), *Proteobacteria* (6.11%, 3.93%, and 4.34%, respectively), and *Fusobacteriota* (2.79%, 3.02%, and 1.96%, respectively). The relative abundance of *Actinobacteriota* in the PPT group was significantly higher than that in the TPB and EPE groups (*p* < 0.05). The relative abundance of Proteobacteria in the TPB group was significantly higher than that in the EPE and PPT groups (*p* < 0.05). Different storage methods influenced the microbial composition of eggshell surfaces at the phylum level. At the genus level (Figure 6B), Lactobacillus, *Romboutsia*, *Bacteroides*, *Ruminococcus_torques_group*, and *Faecalibacterium* were the five dominant genera across treatments. The five dominant genera in the TPB, EPE, and PPT groups were *Lactobacillus* (12.80, 10.31, and 13.23%, respectively), *Romboutsia* (8.65, 17.22, and 7.61%, respectively), *Bacteroides* (6.83, 6.91, and 7.52%, respectively), *Ruminococcus_torques_group* (3.38, 4.46, and 5.79%, respectively), and *Faecalibacterium* (2.49, 2.40, and 3.13%, respectively). *Romboutsia* in the EPE group was significantly more abundant than in the TPB and PPT groups (*p* < 0.05). These findings suggest that storage methods affect the relative abundance of specific bacterial genera on the eggshell surface.

To further visualize the distribution of dominant genera, a ternary plot was constructed (Figure 6C). Lactobacillus was more abundant in the TPB and PPT groups, whereas *Romboutsia* was more abundant in the EPE group. *Bacteroides* and *Faecalibacterium* were evenly distributed among the three groups. Meanwhile, the *Ruminococcus torques group* showed a preference for the PPT. *Turicibacter* and *CHKCI001* were biased toward the EPE and *Muribaculaceae* toward the TPB. Meanwhile, *Olsenella* and *Fusobacterium* were located centrally without a clear preference. Despite the large proportion of shared genera, distinct storage-dependent distribution patterns were observed, which were consistent with the relative abundance analysis.

To identify bacterial taxa that were differentially abundant among the eggshell surface microbiota under different storage conditions, linear discriminant analysis effect size (LEfSe) was performed across multiple taxonomic levels, including phylum, class, order, family, and genus (Figure 6D,E). This analysis identified the distinct dominant taxa within each storage group. In the EPE group, *g__Turicibacter* and *g__Lachnospiraceae_NK4A136_group* were enriched. The TPB group was characterized by higher abundance of *f__Muribaculaceae*, *g__Muribaculaceae*, *g__Bifidobacterium*, *f__Bifidobacteriaceae*, and *o__Bifidobacteriales*. In contrast, the PPT group was dominated by *c__Bacilli, g__Ruminococcus_torques_group*, *g__Corynebacterium*, *f__Corynebacteriaceae*, and *o__Corynebacteriales*. These findings suggest that each storage method affected the abundance of specific taxa and shaped unique microbial signatures.

Genus-level co-occurrence networks were constructed based on the top 100 most abundant genera to further examine the potential interactions among microbes under different storage conditions (Figure 6F–H). Figure 6F–H show the EPE, TPB, and PPT groups, respectively. Spearman correlations (|R| > 0.8, *p* < 0.05) were calculated to assess pairwise relationships among the genera. Densely connected modules were identified using the Louvain algorithm. Compared to the EPE group, the TPB and PPT groups exhibited more complex networks with a greater number of nodes and edges. This indicated stronger microbial interactions under these storage conditions. In contrast, the EPE network was relatively sparse, suggesting weak associations among taxa. Therefore, the storage method shapes the relative abundance of eggshell surface microbiota and influences the complexity and connectivity of microbial community interactions.

### 3.6. Functional Prediction of Eggshell Surface Microbiota

**Figure 7:**
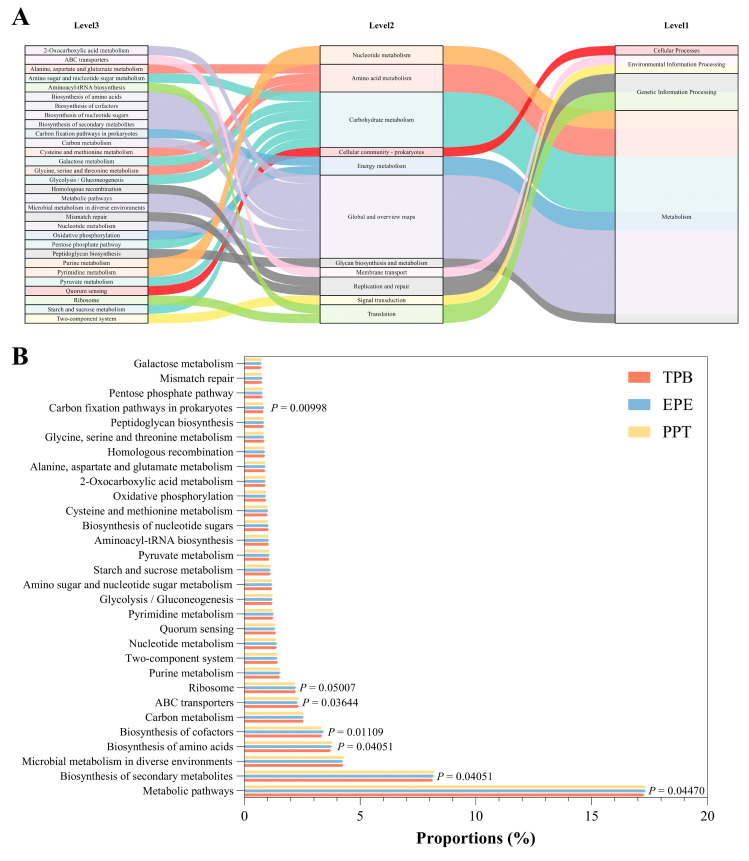
Functional prediction of eggshell surface microbiota based on PICRUSt2 analysis. (**A**) Sankey diagram showing the top 30 KEGG pathways at level 3, grouped by KEGG categories; (**B**) Differential abundance of the top 30 KEGG pathways among EPE, TPB, and PPT groups.

To further investigate the functional implications of the observed shifts in eggshell surface microbiota under different storage conditions, a predictive functional analysis was conducted using PICRUSt2 based on 16S rRNA sequencing data. The top 30 Kyoto Encyclopedia of Genes and Genomes (KEGG) pathways at level 3, ranked by relative abundance, were visualized using a Sankey diagram (Figure 7A). These pathways were distributed across 11 Level 2 and four Level 1 categories. Differential abundance analysis of these pathways showed seven genes that were significantly enriched (*p* < 0.05). This included carbon fixation pathways in prokaryotes, ribosomes, ABC transporters, amino acid biosynthesis, cofactor biosynthesis, secondary metabolite biosynthesis, and metabolic pathways (Figure 7B). Differences in eggshell surface microbiota among storage groups may have functional consequences, particularly in metabolic and biosynthetic processes. This could influence eggshell quality and microbial interactions during storage.

## 4. Discussion

Egg translucency is a common appearance defect in table eggs, which affects consumer acceptance and leads to considerable economic losses [28]. Different packaging materials significantly influenced the development of eggshell translucency during storage. Compared to the TPB and PPT groups, eggs stored in EPE packaging exhibited a lower incidence of translucency. Egg translucency is closely associated with factors such as breed, genetic background, environmental conditions, and eggshell structure [4,16,17]. Shi et al. [4] reported that chronic heat stress significantly increases the proportion of TE. Eggs stored under high-temperature and high-humidity conditions have more severe translucency than those stored in high-temperature and low-humidity environments [29]. Translucency primarily arises from the accumulation of moisture within the eggshell, resulting in the appearance of grayish spots on the shell surface under natural light illumination [12]. After being laid, eggs continuously exchange water vapor and gases with their surrounding environment. External temperature and humidity affect the efficiency of vapor exchange [30]. The translucency trait mainly originates from the absorption and redistribution of moisture within the eggshell matrix and membrane. This alters light refraction and causes translucent spots to appear on the shell surface [12,15,31].

Compared with PPT and TPB, EPE packaging has superior thermal insulation and moisture resistance, which helps stabilize the relative humidity around the eggshell surface. EPE likely reduces the formation of TE by mitigating fluctuations in ambient temperature and humidity, minimizing external environmental effects and restricting moisture penetration and accumulation within the shells. The lower translucency observed in the EPE-packaged eggs may be attributed to their ability to maintain a stable microclimate that minimizes water vapor exchange and internal condensation. Variations in environmental temperature and humidity play critical roles in egg translucency [29,30]. The results of this study highlight the potential industrial value of EPE as an effective packaging material for preserving eggshell appearance and quality during transportation and storage.

Although significant differences in eggshell translucency were observed among the different packaging treatments, no variations were detected in the eggshell microstructure or elemental composition between the TE and NTE groups or between the TA and NTA groups. The uniformity in TT, effective layer, and MT, and the consistent levels of calcium, phosphorus, and magnesium, suggest that translucency is not caused by structural defects or irregular mineral deposition [18,32]. It is more likely attributed to dynamic changes in eggshell moisture and organic matrix properties. A higher matrix protein content in TE may alter the vapor exchange efficiency compared to normal eggs [31]. Increased interspaces between papillae in the mammillary layer enhance water retention, promoting the development of translucent eggs [4,14]. Xuan et al. [15] used micro-CT to visualize the three-dimensional structure of TE and found that they have narrower, irregular, and highly branched pores that are often disconnected from the shell surface. This leads to prolonged moisture retention and reduced vapor exchange efficiency. This influenced the moisture and vapor exchange between eggs and the surrounding environment, rather than differences in eggshell structure or mineral composition. This finding further supports that packaging conditions determine translucency formation by affecting external rather than shell-intrinsic factors.

Egg quality during storage is primarily influenced by storage duration and temperature, and the packaging type plays a contributory role [6,33]. In this study, it was found that changes in packaging materials did not adversely affect the internal quality of eggs. At the same sampling time points, there were no significant differences among the three packaging methods in terms of eggshell strength, thickness, EW, YC, albumin height, or HU. However, with longer storage time, egg quality gradually declined, primarily reflected by a significant reduction in AH and Haugh units after 14 d of storage. Under identical environmental conditions, the packaging type had minimal influence on the internal quality of eggs. These findings are consistent with those of previous studies, which reported that egg quality deteriorates progressively with increasing storage duration [21,34]. Prolonged storage time and elevated temperatures accelerate the decline in egg quality [35]. Jin et al. [36] reported similar trends in the deterioration of the internal quality with increasing temperature and duration. Lu et al. [37] demonstrated that storage duration significantly affected egg quality, as manifested by changes in the sensory characteristics of the albumen and yolk, a decrease in polyunsaturated fatty acids, and an increase in monounsaturated fatty acids. Sokołowicz et al. [9] compared cardboard and plastic packaging and found no significant differences in the albumen foaming capacity or sensory properties of cooked eggs. Eggs stored under vacuum conditions at 22 °C for 42 d maintained higher specific gravity, albumen height, Haugh unit, and yolk index, along with lower weight loss and reduced pH values of both albumen and yolk, compared with conventionally stored eggs [9]. Translucent eggs do not significantly differ from normal eggs in terms of albumen height, yolk color, or Haugh unit values [16,20]. Although some studies have indicated a positive correlation between the rate of water loss and translucency, this appears to be limited to a few specific breeds [17]. Overall, storage duration, rather than packaging material, is the primary factor contributing to a decline in internal egg quality, particularly in terms of albumen freshness and Haugh units.

A rich microbial community on the eggshell surface can profoundly affect egg safety and increase the risk of spoilage. Although factors such as temperature and washing treatments have been extensively investigated, the influence of different storage methods on the composition and diversity of eggshell microbiota remains largely overlooked [21,29,38]. Understanding the microbial community dynamics under various storage conditions is essential for optimizing egg preservation strategies and ensuring food safety. In this study, 16S rRNA sequencing showed distinct differences in the microbial community structures of eggshells under different packaging conditions. The localization, diversity, and composition of eggshell microbiota are shaped by environmental factors, which can, in turn, influence egg quality [39].

Analysis of α- and β-diversity indices showed significant differences in microbial composition and diversity on eggshell surfaces among the three packaging materials (EPE, TPB, and PPT). In the α-diversity analysis, the PPT group showed significantly higher ACE and Chao indices than the EPE group (*p* < 0.05). This indicated that packaging material affects microbial diversity on the eggshell surface. In β-diversity analysis, PCoA showed distinct clustering patterns among the three groups. This suggests that packaging type has a strong influence on the microbial community structure. Zhao et al. [22] used metagenomic approaches to examine microbial community changes in eggshells at different storage times (0, 7, and 14 d) and found that microbial composition changed with prolonged storage. Sokołowicz et al. [9] compared eggs stored in cardboard and plastic boxes and reported that plastic-packaged eggs had higher bacterial loads. This indicated that plastic packaging may favor bacterial proliferation. Vacuum packaging can substantially reduce microbial colonization and extend egg shelf life [38]. De Reu et al. [40] reported that most bacteria detected in eggshells were Gram-positive. In contrast, Gram-negative bacteria penetrate the eggshell and membrane barriers more readily, leading to egg spoilage, although some Gram-positive bacteria may also be present.

Packaging materials significantly influenced the composition. At the phylum level, although *Firmicutes*, *Bacteroidota*, *Actinobacteriota*, *Proteobacteria*, and *Fusobacteriota* dominated all groups, collectively accounting for over 96% of the total relative abundance, their relative proportions varied across packaging types. This suggests that the storage environment selectively shaped the microbial community structure. The dominant phyla across all three packaging treatments were Firmicutes, *Bacteroidota*, and *Actinobacteriota*, which accounted for more than 80% of the total microbiota. These phyla are also predominant in the cecal microbiota of chickens, indicating a potential correlation between the eggshell and maternal gut microbial composition [41]. Compared with the TPB and EPE groups, the PPT group showed significantly higher relative abundance of Actinobacteria (*p* < 0.05). Meanwhile, *Proteobacteria* were significantly enriched in the TPB group. These patterns are consistent with previous studies showing that microclimatic conditions created by packaging, such as humidity, porosity, and oxygen permeability, modulate microbial survival and the composition of eggshells [38]. Porous pulp packaging readily absorbs moisture, favoring the growth of desiccation-tolerant bacteria, such as actinomycetes. Meanwhile, impermeable plastic creates a relatively humid and anaerobic microenvironment conducive to the proliferation of Proteobacteria [42]. Similar packaging-dependent effects have been observed in other food-contact microbiomes where surface physical properties influence microbial adhesion and biofilm formation [9,41]. *Romboutsia* showed significantly higher relative abundance in the EPE group than in the TPB and PPT groups. This genus, which belongs to the family *Peptostreptococcaceae*, is well-known for its fermentative metabolism and adaptability to microaerophilic environments [43]. The prevalence of *Romboutsia* in EPE-packaged eggs indicated that facultative anaerobes gain a selective advantage under partially sealed and moderately humid conditions.

LEfSe analysis showed significantly different taxa associated with storage type. *Turicibacter* and *Lachnospiraceae_NK4A136_group* were significantly enriched in the EPE group. *Turicibacter* is an anaerobic Gram-positive bacterium involved in systemic immune activation that exhibits pro-inflammatory properties, with an increased abundance observed during intestinal inflammation [44]. Cheng et al. [45] reported that *Turicibacter* abundance in the vaginal microbiota of hens producing speckled eggs was significantly higher than in normal hens. The *Lachnospiraceae NK4A136 group* plays a crucial role in maintaining the stability of cecal microbiota. Muribaculaceae and Bifidobacterium were the predominant genera in the TPB group. Jin et al. [46] identified *Muribaculaceae* as a biomarker in 12-d-old egg yolks with a higher prevalence in healthy groups. Bifidobacteria are known to improve laying hen performance [47]. *Ruminococcus torques_group* and Corynebacterium were predominant in the PPT group. Cecal *Ruminococcus* torque abundance negatively correlates with laying hen performance. *Corynebacteriales* and *Bacilli* are resilient environmental bacteria that are typically adapted to dry, oxygen-rich surfaces. This is consistent with the moisture-absorbing properties of pulp trays that maintain egg dryness and aeration [48,49].

Microbial network analysis showed that the TPB and PPT groups exhibited higher node counts and modularity than the EPE group. This indicated more complex interactions among taxa in these samples. Higher network connectivity may reflect more frequent metabolic complementation or competitive interactions. However, it could also render the community more sensitive to external perturbations. By contrast, the EPE group showed a simplified network structure, potentially representing a more stable system dominated by a few robust taxa. This observation aligns with the diversity–stability theory, suggesting that under specific environmental stressors, systems with lower diversity but higher functional complementarity can maintain relative ecological stability [50].

Functional prediction using PICRUSt2 indicated the significant enrichment of seven KEGG level-3 pathways under specific packaging conditions. This included carbon fixation pathways in prokaryotes, ribosomes, ABC transporters, amino acid biosynthesis, cofactor biosynthesis, secondary metabolite biosynthesis, and general metabolic pathways. These enrichments suggest enhanced metabolic diversity and biosynthetic potential under specific packaging conditions. The enrichment of ribosomal and amino acid biosynthesis pathways implies elevated translational activity. Meanwhile, the prevalence of ABC transporters and secondary metabolite biosynthesis indicates active nutrient exchange and potential antimicrobial compound production [51]. These functional differences highlight that packaging materials affect community composition and may modulate metabolic activity and ecological function. PICRUSt2 predictions are based on reference genomes and may not fully reflect in situ metabolic activity. Therefore, validation via metagenomics, metatranscriptomics, or metabolomics is warranted to elucidate the true functional impact of packaging on eggshell microbiota.

From a food safety perspective, these findings indicate that packaging modulates microbial communities, suppressing or facilitating the persistence of spoilage and pathogenic bacteria. Packaging materials that promote a balanced and metabolically active microbiota, such as EPE, may enhance microbial stability and reduce pathogen colonization via competitive exclusion or antimicrobial metabolite production. Future research integrating metagenomics, metatranscriptomics, and targeted metabolomics is necessary to validate these predicted functional potentials and establish causal relationships between packaging microenvironments, microbial ecology, and egg quality during storage.

## 5. Conclusions

In this study, it was demonstrated that the storage method affected the development of egg translucency and composition of the eggshell surface microbiota. Meanwhile, storage duration was the main driver of internal quality deterioration. EPE packaging showed a mitigating effect on translucency compared to PPT and TPB packaging. However, no significant differences in conventional quality traits were observed among the storage methods. Microstructural and elemental analyses indicated that translucency was not linked to shell thickness or mineral content but was likely associated with moisture dynamics. Distinct microbial communities are shaped by different packaging materials. These findings have practical implications for optimizing storage strategies. The optimization of packaging materials to modulate eggshell moisture and microbiota offers a promising strategy for reducing translucency, preserving egg quality, extending shelf life, and ensuring microbial safety during storage. However, it should be noted that this study was conducted under a single storage condition, and microbial functions were predicted based on 16S rRNA data rather than directly validated. Future studies should include multiple environmental conditions and multi-omics approaches to further confirm these findings.

## Figures and Tables

**Table 1:** Effects of different storage methods and storage time on egg quality.

Day	Group	ESI	ESS (kg/cm^2^)	EW (g)	YC	AH (mm)	HU
0	Overall	1.31 ± 0.01	40.72 ± 1.06	64.56 ± 0.77	11.27 ± 0.24	4.75 ± 0.41	71.52 ± 3.09
7	EPE	1.27 ± 0.02	43.10 ± 2.92	65.16 ± 1.56	10.8 ± 0.37	5.31 ± 0.31	78.46 ± 2.09
	PPT	1.27 ± 0.02	43.26 ± 1.71	65.49 ± 1.54	10.9 ± 0.18	4.68 ± 0.45	72.71 ± 3.55
	TPB	1.29 ± 0.01	42.35 ± 2.50	65.22 ± 1.78	10.80 ± 0.20	5.26 ± 0.36	77.78 ± 2.46
	*p*-value	0.625	0.951	0.990	0.932	0.478	0.364
14	EPE	1.28 ± 0.01	44.16 ± 4.08	68.7 ± 1.93	11.60 ± 0.40	2.28 ± 0.31	46.94 ± 7.84
	PPT	1.28 ± 0.01	42.21 ± 1.43	67.93 ± 1.03	11.50 ± 0.31	2.11 ± 0.54	40.81 ± 6.82
	TPB	1.29 ± 0.01	37.36 ± 3.93	66.17 ± 1.29	11.80 ± 0.29	2.29 ± 0.17	43.1 ± 2.86
	*p*-value	0.264	0.344	0.137	0.771	0.251	0.306
21	EPE	1.28 ± 0.02	37.26 ± 4.13	62.72 ± 1.92	12.33 ± 0.33	1.05 ± 0.28	36.90 ± 3.27
	PPT	1.31 ± 0.01	34.27 ± 1.44	64.63 ± 1.20	12.17 ± 0.65	1.23 ± 0.19	31.30 ± 4.09
	TPB	1.28 ± 0.01	38.49 ± 1.83	63.12 ± 1.45	13.2 ± 0.37	1.25 ± 0.29	30.56 ± 4.52
	*p*-value	0.197	0.320	0.622	0.380	0.282	0.901

Data are expressed as mean ± SE. ESI, egg shape index; ESS, eggshell strength; EW, egg weight; YC, yolk color; AH, albumen height; HU, Haugh unit. “Overall” indicates baseline values before storage. *p*-values represent the effects of storage methods at each time point. Differences were considered significant at *p* < 0.05.

## Data Availability

The original contributions presented in this study are included in the article. Further inquiries can be directed to the corresponding author.

## Abbreviations

PPT	Paper pulp tray
EPE	Expanded polyethylene foam tray
TPB	Transparent plastic box
TT	Total thickness
ET	Effective layer thickness
MT	Mammillary layer thickness
EMT	Eggshell membrane thickness
TE	translucent egg
NTE,	Non-translucent egg
NTA	Non-translucent area
TA	Translucent area
ESI	Egg shape index
EW	Egg weight
ESS	Eggshell strength
AH	Egg white height
HU	Haugh units
YC	Yolk color
ASV	An amplicon sequence variant
PCoA	Principal coordinate analysis
LEfSe	Linear discriminant analysis effect size
KEGG	Kyoto Encyclopedia of Genes and Genomes
ANOVA	Analyzed using one-way analysis of variance
